# Interference Management for Adaptive Full-Duplex V2V Communications with Delayed CSI Feedback

**DOI:** 10.3390/s26196128

**Published:** 2026-09-27

**Authors:** Yuetian Zhou, Cheng Yan, Yahao Wang, Yang Li, Chang Liu, Shihai Shao

**Affiliations:** 1 National Key Laboratory of Wireless Communications, University of Electronic Science and Technology of China, Chengdu 611731, China; 202212220621@std.uestc.edu.cn (Y.Z.); 202312220628@std.uestc.edu.cn (C.Y.); 202521220411@std.uestc.edu.cn (Y.W.); ly1999@std.uestc.edu.cn (Y.L.); 202312220629@std.uestc.edu.cn (C.L.); 2 Future Technology Research Center, China Telecom Research Institute, Beijing 102209, China

**Keywords:** adaptive full-duplex, CSI feedback delay, full-duplex V2V communications, Kuhn–Munkres algorithm

## Abstract

This paper addresses the performance degradation caused by delayed channel state information (CSI) feedback in vehicular networks by introducing full-duplex (FD) technology into vehicle-to-vehicle (V2V) communications. We jointly optimize power control and spectrum allocation, designing an FD-V2V interference management algorithm aimed at maximizing the ergodic sum rate and the minimum ergodic rate of vehicle-to-infrastructure (V2I) links, respectively. Furthermore, an adaptive full-duplex V2V (AFD-V2V) interference management algorithm is proposed to dynamically select the optimal duplex mode. The proposed algorithm maximizes system performance while guaranteeing V2V link reliability by integrating large-scale fading information, statistical properties of small-scale fading, and delayed CSI of small-scale fading from non-directly connected base station links. The simulation results demonstrate that the AFD-V2V scheme significantly outperforms traditional half-duplex V2V (HD-V2V) in both the ergodic sum rate and the minimum ergodic rate.

## 1. Introduction

The Internet of vehicles (IoV) is an important application scenario of future 6G wireless networks, where vehicles require reliable and low-latency communication under highly dynamic channel and interference conditions [1]. With the increasing number of connected vehicles and the scarcity of spectrum resources, spectrum sharing between vehicle-to-vehicle (V2V) links and uplink vehicle-to-infrastructure (V2I) links has become a key technique for improving spectrum efficiency [2]. However, such spectrum sharing also creates a coupled interference management problem, where V2I throughput and V2V reliability must be jointly considered. Recent flow-aware studies further indicate that mobile service demand and network resource states evolve continuously over space and time [3,4]. This evolution motivates adaptive resource management in highly dynamic wireless networks. This spatiotemporal perspective naturally calls for resource allocation schemes that adapt to both CSI aging and interference dynamics, which are the two key challenges addressed in this work.

A major difficulty in vehicular resource allocation lies in the acquisition of reliable channel state information (CSI). Due to high vehicle mobility, instantaneous small-scale CSI becomes outdated rapidly, especially for links that are not directly connected to the base station (BS). Relying only on large-scale fading and small-scale fading statistics improves implementability. However, this approach may forgo useful delayed CSI observations, potentially causing performance loss. On the other hand, assuming perfect instantaneous CSI can lead to overly optimistic designs that are difficult to realize in practical IoV scenarios.

To balance performance and implementability, delayed-CSI-aware interference management provides a practical technical route [5]. In this route, the BS uses large-scale CSI, statistical small-scale fading information, and delayed small-scale CSI of non-directly connected links to optimize resource allocation. Full-duplex (FD) V2V communication is particularly attractive in this context because bidirectional concurrent transmission can reduce transceiver-switching latency. In the considered feedback architecture, this bidirectional capability can also shorten the effective CSI reporting delay. Nevertheless, FD operation introduces residual self-interference and stronger mode-dependent interference coupling [6]. Therefore, an adaptive FD/half-duplex (HD) mode selection mechanism is needed so that FD is activated only when its bidirectional transmission gain outweighs its interference penalty [7].

### 1.1. Related Work

The related literature can be organized along three research dimensions: (i) duplex mode and CSI modeling, (ii) resource allocation methods, and (iii) solution methodology.

In the first dimension, resource allocation for vehicle-to-everything (V2X) and sidelink communication has been widely investigated. Recent surveys have summarized mathematical formulations, standardization trends, and open challenges in 6G V2X sidelink resource allocation [1,8]. Classical V2V/V2I spectrum sharing studies considered cellular uplink resources reused by V2V pairs and formulated joint spectrum and power allocation problems under V2V reliability constraints [2,9]. In particular, large-scale-fading-based optimization reduces the dependence on instantaneous CSI and is therefore suitable for high-mobility vehicular environments [2]. Ergodic-capacity-based user pairing and power control further exploit statistical small-scale fading information to improve long-term system performance [9]. Game-theoretic and learning-based methods have also been applied to dynamic V2X resource allocation, enabling more flexible adaptation to changing interference and mobility conditions [10,11,12,13].

In the second dimension, an important line of work focuses on imperfect, outdated, or delayed CSI. Delayed CSI feedback was explicitly considered in device-to-device (D2D)-enabled vehicular communications, where the BS receives periodically reported CSI from V2V links and optimizes spectrum and power allocation accordingly [5]. Related studies also investigated outdated-CSI-based power allocation in cooperative V2V systems [14], robust Stackelberg-game-based allocation in D2D-enabled vehicular networks [15], and one-to-many D2D-based vehicular communication with delayed CSI feedback [16]. Imperfect-CSI-based spectrum and power allocation has also been investigated in V2X and underlay D2D networks [17,18]. These studies consistently show that CSI aging degrades either V2I capacity or V2V throughput and that delay-aware resource allocation is necessary in high-mobility networks. However, most delayed-CSI vehicular studies are built upon half-duplex (HD) V2V communication, where the potential latency benefit of FD bidirectional transmission is not exploited.

In the third dimension, FD communication has attracted increasing attention because it can support simultaneous transmission and reception over the same spectrum. Practical self-interference cancellation (SIC) prototypes have demonstrated that high SIC capability is achievable, providing a physical basis for FD deployment [6]. In vehicular networks, FD discovery and resource distribution have been studied for 5G-V2X systems [19], and FD mechanisms have also been considered in mmWave vehicular content distribution [20]. In parallel, FD-D2D resource allocation under cellular networks has been investigated through graph-based or optimization-based methods [21,22]. More recently, adaptive mode switching under imperfect CSI has been explored in D2D networks for automated guided vehicle (AGV)-based factory automation [7]. However, that work targets indoor low-mobility AGV scenarios with generic imperfect CSI, whereas this paper addresses outdoor high-mobility vehicular networks with explicit delayed CSI modeling and the FD delay coefficient κ. Albataineh et al. [23] studied adaptive duplexing for decentralized FD-non-orthogonal multiple access (NOMA) V2X systems; however, that work adopts NOMA with a decentralized architecture and Monte Carlo simulation, whereas this paper considers orthogonal multiple access (OMA)-based centralized allocation with closed-form analytical solutions under delayed CSI.

A dedicated line of research has investigated FD transceivers in V2X sidelink communications. Bazzi et al. [24] enhanced cooperative driving over IEEE 802.11 vehicular networks by exploiting FD radios, which enable vehicles to detect collisions while transmitting. Campolo et al. [25] extended FD capabilities to the 5G-V2X sidelink autonomous mode, and Todisco et al. [26] further proposed FD-based collision detection with adaptive thresholds and resource reselection for the NR sidelink autonomous mode. These studies demonstrate the practical benefits of FD in distributed contention-based vehicular environments. In contrast, this paper considers a centralized cellular underlay architecture in which the BS performs closed-form power control and bipartite spectrum matching under delayed CSI feedback. The two lines of work are complementary: the FD sidelink studies address collision detection and resource reselection among autonomously scheduling vehicles, whereas the interference coupling between V2I and V2V links and the impact of CSI aging considered in this paper cannot be handled by distributed contention mechanisms and instead require centralized optimization.

To the best of our knowledge, no existing work has jointly addressed delayed CSI feedback and FD bidirectional transmission in V2V communications. The interplay between CSI aging reduction—enabled by the FD delay coefficient κ—and FD-specific interference management remains unexplored, which is precisely the gap this paper fills. Nevertheless, existing FD-related studies either focus on non-vehicular D2D scenarios, assume ideal or perfect CSI, or do not jointly address delayed CSI feedback, V2V reliability, FD/HD mode selection, and multi-user spectrum allocation in a unified FD-V2V framework.

Table 1 provides a systematic comparison of the most closely related works along the dimensions of duplex mode, CSI availability, optimization objective, and solution method. These works are selected because each represents a distinct combination of duplex mode and CSI modeling that differs from this paper in at least one key dimension; the remaining cited works either share the same combination as one of the selected references or provide background without proposing a specific resource allocation method.

### 1.2. Contributions and Organization

Motivated by the above gap, this paper studies interference management for adaptive full-duplex V2V communications with delayed CSI feedback. The BS is assumed to know the large-scale CSI of all the links, the statistical distribution of small-scale fading, and the delayed small-scale CSI of links that are not directly connected to the BS. Under this practical information structure, power control, spectrum allocation, and FD/HD mode selection are jointly considered. The main contributions of this paper are summarized as follows. Although the Kuhn–Munkres (KM) matching algorithm and outage probability formulation are established tools, the novelty of this work lies not in these tools themselves but in the new theoretical results they enable: (i) a joint optimization framework for delayed CSI and FD bidirectional transmission, (ii) closed-form analytical solutions for the FD-V2V ergodic rate, feasible region, and optimal power, and (iii) a bidirectional outage constraint structure that is specific to FD-V2V and has no counterpart in HD-based studies.

- We develop a delayed-CSI-aware interference management framework for adaptive full-duplex V2V communications with cellular uplink spectrum reuse. Unlike existing HD-oriented delayed-CSI models, the proposed framework jointly captures bidirectional FD transmission, residual self-interference, cellular user vehicle (CUV)-to-D2D user vehicle (DUV) interference, and CSI aging within a unified optimization model. The resulting reliability constraint is characterized by the simultaneous outage behavior of the two transmission directions.
- We derive a tractable power-control solution for each candidate CUV–DUV sharing pair under delayed CSI feedback. Closed-form expressions for the ergodic V2I rate and the outage-constrained feasible transmit-power region are obtained. These expressions enable the BS to efficiently construct a rate matrix by combining large-scale CSI, statistical channel information, and delayed small-scale CSI.
- Building upon the pairwise optimization results, we develop matching-based spectrum allocation algorithms under both sum rate and max–min objectives together with an adaptive FD/HD (AFD) mode selection mechanism. The proposed scheme reveals that the performance gain of adaptive full-duplex originates from the complementarity between FD and HD under different channel, interference, CSI-delay, and self-interference conditions. That is, the gain arises from adaptive selection rather than universally preferring one duplex mode.

The remainder of this paper is organized as follows. Section 2 presents the system model and formulates the delayed-CSI-aware FD-V2V resource allocation problems. Section 3 derives the single-pair power control solution and develops the multi-user spectrum allocation and adaptive duplex mode selection algorithms. Section 4 provides the numerical results and discusses the impact of CSI delay, vehicle mobility, and SIC capability. Section 5 concludes this paper.

## 2. System Model and Problem Formulation

This section presents the networking model and formulates the corresponding optimization problems aimed at maximizing the ergodic sum rate and minimum rate of V2I, respectively. The detailed mathematical formulation is provided only for the FD-V2V sharing mode; the HD-V2V derivation follows a parallel procedure, and interested readers are referred to [2] for the single-direction outage constraint formulation.

### 2.1. System Model

The system model for FD-V2V and HD-V2V underlying a cellular network is illustrated in Figure 1. It is assumed that the cell contains *J* cellular user vehicles (cuvs), denoted as CUV_*j*_, and *I* D2D user vehicle (DUV) pairs, denoted as DUV_*i*_, where j∈C={1,2,…,J} and i∈D={1,2,…,I}. To facilitate the acquisition of CSI at the BS, the communication in each DUV pair typically reuses the uplink spectrum of one CUV.

The main symbols used throughout this paper are summarized in Table 2.

The channel model in this paper incorporates path loss, shadow fading, and small-scale fast fading. Taking the channel $g_{j,B}$ from CUV_*j*_ to the BS as an example, the channel power gain is modeled as

(1) $$g_{j,B}=\alpha_{j,B}h_{j,B},$$

where $h_{j,B}={\left|h_{j,B}^{\prime }\right|}^{2}$ is the small-scale fading power, following an exponential distribution with unit mean; $h_{j,B}^{\prime }$ is the small-scale fading amplitude component, following a complex Gaussian distribution; and $\alpha_{j,B}=A\psi_{j,B}d_{j,B}^{-\gamma }$ is the large-scale fading component, encompassing both path loss and shadow fading, with *A* being the path loss constant, γ the path loss exponent, $d_{j,B}$ the distance between CUV_*j*_ and the BS, and $\psi_{j,B}$ the shadow fading random variable following a log-normal distribution with standard deviation ξ. The notations for the remaining channels are defined in a manner consistent with Equation (1), as illustrated in Figure 1.

**Notation Convention:** Throughout this paper, subscript *j* indexes CUVs (j∈C), while subscript *i* indexes DUV pairs (i∈D). For each DUV pair *i*, the two constituent vehicles are denoted as $i_{1}$ (transmitter) and $i_{2}$ (receiver) in the context of a specific transmission direction.

Since small-scale fading experiences rapid variations and cannot be acquired accurately, this paper assumes that the BS can only accurately obtain the slowly varying large-scale fading components. The CSI for links not directly connected to the BS needs to be reported periodically by the DUVs, and the small-scale fading component within this CSI must account for the feedback delay. In the conventional HD-V2V mode, the CSI for links not directly connected to the BS is reported to the BS by the receiver in the DUV pair with a period of *T*, which involves switching time for transmitting and receiving. In contrast, FD-V2V, leveraging its bidirectional transmission capability, can report to the BS instantaneously, significantly reducing the reporting delay. The reporting period for the FD-V2V mode is $T_{\mathrm{FD}}=\kappa T$, where κ is the FD delay coefficient. To balance signaling overhead and delay, κ is typically set between 0.1 and 0.5, while, in HD mode, κ=1. This paper employs a first-order Gauss–Markov process to model the variation in small-scale fading over a period τ for channels not directly connected to the BS [5]:

(2) $$h^{\prime }=\epsilon {\hat{h}}^{\prime }+e=J_{0}\left(2\pi f_{d}kT\right){\hat{h}}^{\prime }+e,$$

where $h^{\prime }$ and ${\hat{h}}^{\prime }$ denote the small-scale fading amplitude components at the current and previous time instances, respectively; the coefficient ϵ quantifies the correlation of the fast fading between two consecutive time slots, which is calculated by the zero-order Bessel function of the first kind, where $f_{d}=vf_{c}/c$, *v* is the vehicle velocity, $f_{c}$ is the carrier frequency, and *c* is the velocity of light; *e* is the innovation term of the channel, following a complex Gaussian distribution $\mathrm{CN}(0,1-\epsilon^{2})$.

**Remark on Channel Model Simplification:** From Equation (2), the squared modulus of the small-scale fading amplitude is |h′|2=ϵ2|h^′|2+|e|2+2ϵRe{h^′e*}. Since ${\hat{h}}^{\prime }$ and *e* are independent zero-mean complex Gaussian random variables, the cross-term 2ϵRe{h^′e*} is a zero-mean random variable whose variance is negligible compared with the dominant terms $\epsilon^{2}{\left|{\hat{h}}^{\prime }\right|}^{2}$ and ${\left|e\right|}^{2}$ in the context of outage probability analysis. Therefore, following the approach in [5], the subsequent SINR expressions in Equations (4) and (5) drop the cross-term.

Consider a specific sharing pair where DUV_*i*_ (with transmitter $i_{1}$ and receiver $i_{2}$) reuses the uplink spectrum of CUV_*j*_. The signal-to-interference-plus-noise ratio (SINR) expressions for the three involved links are given in Equations (3)–(5) as follows:

(3) $$\gamma_{j}^{\mathrm{FD},c}=\frac{P_{j}^{c}\alpha_{j,B}{\left|h_{j,B}^{\prime }\right|}^{2}}{\sigma^{2}+\sum_{i=1}^{I}\rho_{i,j}\left(P_{i}^{d}\alpha_{i_{1},B}|h_{i_{1},B}^{\prime }{|}^{2}+P_{i}^{d}\alpha_{i_{2},B}{\left|h_{i_{2},B}^{\prime }\right|}^{2}\right)},$$

where $i_{1}$ and $i_{2}$ denote the two vehicles in DUV_*i*_, with $i_{1}$ being the transmitter and $i_{2}$ the receiver in the considered direction. The two DUV interference terms in the denominator of Equation (3) reflect that both vehicles of DUV_*i*_ transmit simultaneously in FD mode, whereas only a single term exists in HD mode.

(4) $$\gamma_{i_{1}}^{\mathrm{FD},d}=\frac{P_{i}^{d}\alpha_{i}\left(\epsilon_{i}^{2}|{\hat{h}}_{i}^{\prime }{|}^{2}+{\left|e_{i}\right|}^{2}\right)}{\begin{array}{cc} \; & \sigma^{2}+P_{i}^{d}\cdot \eta +\sum_{j=1}^{J}\rho_{i,j}P_{j}^{c}\alpha_{j,i_{1}}\left(\epsilon_{j,i_{1}}^{2}|{\hat{h}}_{j,i_{1}}^{\prime }{|}^{2}+{\left|e_{j,i_{1}}\right|}^{2}\right) \end{array}},$$

(5) $$\gamma_{i_{2}}^{\mathrm{FD},d}=\frac{P_{i}^{d}\alpha_{i}\left(\epsilon_{i}^{2}|{\hat{h}}_{i}^{\prime }{|}^{2}+{\left|e_{i}\right|}^{2}\right)}{\begin{array}{cc} \; & \sigma^{2}+P_{i}^{d}\cdot \eta +\sum_{j=1}^{J}\rho_{i,j}P_{j}^{c}\alpha_{j,i_{2}}\left(\epsilon_{j,i_{2}}^{2}|{\hat{h}}_{j,i_{2}}^{\prime }{|}^{2}+{\left|e_{j,i_{2}}\right|}^{2}\right) \end{array}},$$

Note that Equations (4) and (5) represent the SINRs at the two respective receivers ($i_{1}$ and $i_{2}$) of the bidirectional FD-V2V link, where $P_{j}^{c}$ is the transmit power of CUV_*j*_, $P_{i}^{d}$ is the transmit power of DUV_*i*_, $\sigma^{2}$ denotes the noise power, η represents the SIC capability in FD mode, and $\rho_{i,j}$ is the user pairing indicator. In Equations (4) and (5), the numerator consists of the conditional-mean part $\epsilon_{i}^{2}{\left|{\hat{h}}_{i}^{\prime }\right|}^{2}$ known from the delayed CSI and the innovation part $|e_{i}{|}^{2}$, and the identical transmit power in the two directions is justified by the reciprocity of the bidirectional link. Specifically, $\rho_{i,j}=1$ when DUV_*i*_ reuses the spectrum with CUV_*j*_ and $\rho_{i,j}=0$ otherwise. Consequently, when CUV_*j*_ shares the spectrum with a potential DUV in FD mode, the ergodic rate of CUV_*j*_ is given by

(6) $$R_{j}^{\mathrm{FD},c}=E\left[\log_{2}\left(1+\gamma_{j}^{\mathrm{FD},c}\right)\right],$$

where E[·] denotes the expectation over the fast-fading random variables. The ergodic rate is the result of averaging over the fast-fading variations to obtain a long-term average.

### 2.2. Problem Formulation

Given that V2I links pursue high data rates while V2V links require high reliability in vehicular networks, this paper formulates optimization problem P1. The objective is to maximize the ergodic sum rate of CUVs while ensuring the reliability of V2V links, with a quality of service (QoS) requirement set for each CUV. V2V link reliability is ensured by controlling the outage probability below a system-tolerable threshold. P1 is modeled as follows:

(7a)P1:maxρi,j,Pjc,Pid∑j=1JRjFD,c(7b)s.t.C1:E[log2(1+γjFD,c)]≥R0c,∀j∈C(7c)C2:Pr{γi1FD,d≤γ0d∩γi2FD,d≤γ0d}≤p0,∀i∈D(7d)C3:0≤Pjc≤Pmaxc,∀j∈C(7e)C4:0≤Pid≤Pmaxd,∀i∈D(7f)C5:∑i=1Iρi,j≤1,ρi,j∈{0,1},∀j∈C(7g)C6:∑j=1Jρi,j≤1,ρi,j∈{0,1},∀i∈D.

where $R_{0}^{c}$ denotes the QoS requirement, $\gamma_{0}^{d}$ is the threshold for V2V link outage, $p_{0}$ represents the tolerable outage probability, and $P_{\max}^{c}$ and $P_{\max}^{d}$ are the maximum transmit powers for CUVs and DUVs, respectively. Constraint C2 ensures that the bidirectional links in FD do not fail simultaneously, i.e., the probability of both directions being in outage does not exceed $p_{0}$, while constraints C5 and C6 define the one-to-one spectrum sharing between CUVs and DUVs.

Besides the sum rate, the minimum rate is another key metric, often reflecting user fairness in the system. Thus, we formulate optimization problem P2 in Equation (8) to maximize the minimum ergodic rate among all users, which is modeled as:

(8) $$\begin{array}{cc} P2:\;\;\;\; & \max_{\rho_{i,j},P_{j}^{c},P_{i}^{d}}\min_{j\in C}R_{j}^{\mathrm{FD},c}\\ s.t.\;\;\;\; & \mathrm{Equations}\;(7b)–(7g). \end{array}$$

Both P1 and P2 are highly non-convex optimization problems for which closed-form solutions are mathematically intractable. This paper will develop efficient and robust interference management algorithms to solve them.

## 3. Interference Management Algorithm

In this section, the solutions to problems P1 and P2 are decoupled into two sequential steps: power control and spectrum allocation. Both P1 and P2 first require determining the optimal power solution that maximizes the ergodic rate of the CUV within a single CUV–DUV sharing pair. Subsequently, distinct spectrum allocation algorithms are employed to perform user pairing between CUVs and DUVs under different optimization objectives. Furthermore, mode selection is incorporated, leading to the proposal of the AFD scheme.

The optimality of this decomposition is formally established in Proposition 1.

**Proposition 1.**  *Under the assumptions that (S1) each CUV–DUV pair occupies an independent spectrum block with no inter-pair interference, (S2) the sum rate (resp. minimum rate) objective is decomposable as a sum (resp. minimum) of per-pair rates, and (S3) each pair’s rate depends only on that pair’s power allocation. The decomposition of P1 (or P2) into pairwise power optimization followed by maximum-weight matching yields the global optimum.*

**Proof.**  Under (S1), the rate $R_{i,j}$ of pair (i,j) depends only on $\left(P_{i}^{d},P_{j}^{c}\right)$. The per-pair optimal rate $R_{i,j}^{*}$ is obtained by Theorem 1. Under (S2), sum rate maximization becomes $\max_{M}\sum_{(i,j)\in M}R_{i,j}^{*}$, which is a maximum-weight bipartite matching problem solved optimally by the KM algorithm in $O(J^{3})$ time. For the MaxMin objective (P2), binary search with feasibility checking via the Hungarian algorithm (Algorithm 1) similarly yields the global optimum. Since both the per-pair power optimization and the matching are individually optimal, their composition is globally optimal.    □

**Algorithm 1** FD-V2V MaxMin Spectrum Allocation**Require:** Ergodic rate matrix $R^{\mathrm{FD}}={\left\{R_{i,j}^{\mathrm{FD},c*}\right\}}_{J\times J}$
**Ensure:** Spectrum sharing matrix $\rho ={\left\{\rho_{i,j}\right\}}_{J\times J}$ and the maximized minimum CUV rate $R_{\min}^{*}$
 1:Sort all elements of $R^{\mathrm{FD}}$ in ascending order and store them in s. 2:Initialize a=1, $b=J^{2}$, and $\rho =0_{J\times J}$. 3:**while** b−a>1 **do** 4:   m=⌊(a+b)/2⌋. 5:   Initialize the judging matrix $E={\left\{e_{i,j}\right\}}_{J\times J}$. 6:   **for** j=1 to *J* **do** 7:     **for** i=1 to *J* **do** 8:        **if** $R_{i,j}^{\mathrm{FD},c*}<s_{m}$ **then** 9:          $e_{i,j}=1$.10:        **else**11:          $e_{i,j}=0$.12:        **end if**13:     **end for**14:   **end for**15:   Apply the Hungarian algorithm to E and obtain the assignment matrix F and the minimum total weight *w*.16:   **if** w>0 **then**17:     b=m.18:   **else**19:     a=m and ρ=F.20:   **end if**21:**end while**22:$R_{\min}^{*}=\min_{j\in C}\sum_{i=1}^{J}\rho_{i,j}R_{i,j}^{\mathrm{FD},c*}$.23:**return** ρ and $R_{\min}^{*}$.


### 3.1. Power Control for Single Sharing Pair

The ergodic rate maximization for CUV_*j*_ sharing spectrum with DUV_*i*_ is formulated as optimization problem P3 in Equation (9):

(9) $$\begin{array}{cc} P3:\;\;\;\; & \max_{P_{j}^{c},P_{i}^{d}}R_{i,j}^{\mathrm{FD},c}\\ s.t.\;\;\;\; & \mathrm{Equations}\;(7c)–(7e). \end{array}$$

The closed-form expression for the objective function of Problem P3 is first derived in the following lemma.

**Lemma 1.**  *The closed-form expression for the ergodic rate $R_{i,j}^{\mathrm{FD},c}$ in the FD-V2V sharing pair CUV_j_–DUV_i_ is given as follows:*

(10) $$\begin{array}{c} R_{i,j}^{\mathrm{FD},c}\left(P_{i}^{d},P_{j}^{c}\right)=\frac{1}{\ln2}\left[\frac{a^{2}E_{1}\left(\frac{1}{a}\right)e^{\frac{1}{a}}}{\left(a-b)(a-c\right)}+\frac{abE_{1}\left(\frac{1}{b}\right)e^{\frac{1}{b}}}{\left(b-a)(b-c\right)}+\frac{acE_{1}\left(\frac{1}{c}\right)e^{\frac{1}{c}}}{\left(c-a)(c-b\right)}\right], \end{array}$$

*where $a=\frac{P_{j}^{c}\alpha_{j,B}}{\sigma^{2}}$, $b=\frac{P_{i}^{d}\alpha_{i_{1},B}}{\sigma^{2}}$, $c=\frac{P_{i}^{d}\alpha_{i_{2},B}}{\sigma^{2}}$, $E_{1}\left(x\right)=\int_{x}^{\infty }\frac{e^{-t}}{t}\;dt$ is the first-order exponential integral function.*

**Proof.**  Denote the random variables $h_{j,B}$, $h_{i_{1},B}$, and $h_{i_{2},B}$ as *X*, $Y_{1}$, and $Y_{2}$, respectively. Since the small-scale fading powers are exponentially distributed with unit mean, *X*, $Y_{1}$, and $Y_{2}$ are independent unit-mean exponential random variables. Let $Z=\frac{aX}{1+bY_{1}+cY_{2}}$. Then, the ergodic rate $R_{i,j}^{\mathrm{FD},c}$ can be expressed as:

(11) $$\begin{array}{cc} R_{i,j}^{\mathrm{FD},c}\left(P_{i}^{d},P_{j}^{c}\right)\; & =E\left[\log_{2}\left(1+\frac{P_{j}^{c}\alpha_{j,B}h_{j,B}}{\sigma^{2}+P_{i}^{d}\alpha_{i_{1},B}h_{i_{1},B}+P_{i}^{d}\alpha_{i_{2},B}h_{i_{2},B}}\right)\right]\\ \; & ≜E\left[\log_{2}\left(1+\frac{aX}{1+bY_{1}+cY_{2}}\right)\right]\\ \; & ≜E\left[\log_{2}\left(1+Z\right)\right]. \end{array}$$

The cumulative distribution function (CDF) of *Z* in Equation (11) is obtained by conditioning on $Y_{1}$ and $Y_{2}$. For a given pair $\left(Y_{1},Y_{2}\right)$ and any z≥0, the event Z≤z is equivalent to $X\leq z(1+bY_{1}+cY_{2})/a$. Hence

(12) $$\begin{array}{cc} F_{Z}\left(z\right) & =\mathrm{Pr}\;\left\{\frac{aX}{1+bY_{1}+cY_{2}}\leq z\right\}\\ \; & =E_{Y_{1},Y_{2}}\;\left[\mathrm{Pr}\;\left\{X\leq \frac{z(1+bY_{1}+cY_{2})}{a}|Y_{1},Y_{2}\right\}\right]\\ \; & =1-E_{Y_{1},Y_{2}}\;\left[\exp\;\left(-\frac{z(1+bY_{1}+cY_{2})}{a}\right)\right]\\ \; & =1-e^{-\frac{z}{a}}E\;\left[e^{-\frac{zbY_{1}}{a}}\right]E\;\left[e^{-\frac{zcY_{2}}{a}}\right]\\ \; & =1-\frac{a^{2}e^{-\frac{z}{a}}}{\left(a+zb)(a+zc\right)}. \end{array}$$

Next, the expectation in Equation (11) can be transformed by integration by parts, as shown in Equation (13). Let u=ln(1+z) and $dv=f_{Z}\left(z\right)dz$. Taking $v=F_{Z}\left(z\right)-1$ avoids a nonzero boundary term at infinity and gives

(13) $$\begin{array}{cc} \int_{0}^{\infty }\ln\left(1+z\right)f_{Z}\left(z\right)dz & ={\left[\ln\left(1+z\right)\left(F_{Z}\left(z\right)-1\right)\right]}_{0}^{\infty }+\int_{0}^{\infty }\frac{1-F_{Z}\left(z\right)}{1+z}dz. \end{array}$$

The boundary term is zero: it is zero at z=0, and, as z→∞, Equation (12) gives $1-F_{Z}\left(z\right)=\frac{a^{2}e^{-z/a}}{\left(a+zb)(a+zc\right)}$, whose exponential decay dominates the logarithmic growth of ln(1+z). Therefore,

(14) $$\begin{array}{cc} R_{i,j}^{\mathrm{FD},c} & =\frac{1}{\ln2}\int_{0}^{\infty }\ln\left(1+z\right)f_{Z}\left(z\right)dz\\ \; & =\frac{1}{\ln2}\int_{0}^{\infty }\frac{1-F_{Z}\left(z\right)}{1+z}dz\\ \; & =\frac{1}{\ln2}\int_{0}^{\infty }\frac{a^{2}e^{-\frac{z}{a}}}{\left(1+z)(a+zb)(a+zc\right)}dz. \end{array}$$

The remaining integral can be evaluated by partial fraction decomposition as in Equation (15),

(15) $$\frac{a^{2}}{\left(1+z)(a+zb)(a+zc\right)}=\frac{A}{1+z}+\frac{B}{a+zb}+\frac{C}{a+zc},$$

where *A*, *B*, and *C* are constants determined by *a*, *b*, and *c*. Each term is then reduced to the standard integral $\int_{0}^{\infty }\frac{e^{-z/a}}{z+\varphi }dz=e^{\varphi /a}E_{1}\left(\varphi /a\right)$. Substituting the corresponding coefficients gives Equation (10). □

**Remark on the degenerate case of Equation (10):** When b=c, Equation (10) involves a removable singularity, and the degenerate closed-form is obtained via partial fraction decomposition with a repeated pole ${\left(a+zb\right)}^{-2}$:

(16) $$R_{i,j}^{\mathrm{FD},c}=\frac{1}{\ln2}\left[\frac{a^{2}\left(e^{1/a}E_{1}\left(1/a\right)-e^{1/b}E_{1}\left(1/b\right)\right)}{{\left(a-b\right)}^{2}}+\frac{a\left(\frac{1}{b}e^{1/b}E_{1}\left(1/b\right)-1\right)}{a-b}\right].$$

The cases a=b and a=c are handled analogously. In numerical implementation, Equation (16) is used when |b−c| falls below a threshold to avoid catastrophic cancellation in Equation (10).

Lemma 1 indicates that, when the BS has knowledge of the large-scale fading, the optimization objective becomes a function of the transmit powers $P_{i}^{d}$ and $P_{j}^{c}$, with its feasible domain determined by constraints C2–C4 in problem P1, while Lemma 2 specifies the corresponding conservative feasible region.

**Lemma 2.**  *The conservative feasible region of the transmit powers for the FD-V2V sharing pair CUV_j_–DUV_i_ is given by the following two sets:*

(17) $$\left\{\left(P_{i}^{d},P_{j}^{c}\right)\;|\;\begin{array}{c} \exp\left(\frac{G\gamma_{0}^{d}}{F}\right)\left(1+\frac{H}{F}\gamma_{0}^{d}\right)\leq \frac{\exp(E/F)}{1-p_{1}},\\ G\gamma_{0}^{d}\geq E,\;0\leq P_{j}^{c}\leq P_{\max}^{c},\;0\leq P_{i}^{d}\leq P_{\max}^{d} \end{array}\right\},$$

(18) $$\left\{\left(P_{i}^{d},P_{j}^{c}\right)\;|\;\begin{array}{c} \left(1+\frac{F}{\gamma_{0}^{d}H}\right)\exp\left(\frac{E-G\gamma_{0}^{d}}{\gamma_{0}^{d}H}\right)\geq \frac{1}{p_{1}},\\ G\gamma_{0}^{d}<E,\;0\leq P_{j}^{c}\leq P_{\max}^{c},\;0\leq P_{i}^{d}\leq P_{\max}^{d} \end{array}\right\}.$$

*where $E=P_{i}^{d}\alpha_{i}\epsilon_{i}^{2}{\left|{\hat{h}}_{i}^{\prime }\right|}^{2}$, $F=P_{i}^{d}\alpha_{i}\left(1-\epsilon_{i}^{2}\right)$, $G=\sigma^{2}+P_{i}^{d}\eta +P_{j}^{c}\alpha_{j,i}\epsilon_{j,i}^{2}{\left|{\hat{h}}_{j,i}^{\prime }\right|}^{2}$, and $H=P_{j}^{c}\alpha_{j,i}\left(1-\epsilon_{j,i}^{2}\right)$.*

**Proof.**  By virtue of the independent fading assumption stated after Equation (7c), the two outage events in C2 are independent, and C2 reduces to the product of marginal probabilities $\mathrm{Pr}\left\{\gamma_{i_{1}}^{\mathrm{FD},d}\leq \gamma_{0}^{d}\right\}\cdot \mathrm{Pr}\left\{\gamma_{i_{2}}^{\mathrm{FD},d}\leq \gamma_{0}^{d}\right\}\leq p_{0}$. Furthermore, the two vehicles in the same DUV pair communicate over a short range, while spectrum allocation aims to pair each DUV with a distant CUV to reduce mutual interference. Consequently, the interference channels from CUV_*j*_ to the two DUV receivers have nearly identical large-scale fading, i.e., $g_{j,i_{1}}\approx g_{j,i_{2}}≜g_{j,i}$. Here, $g_{j,i_{1}}\approx g_{j,i_{2}}$ reflects a modeling equivalence: the BS treats both directions as sharing the same deterministic parameters (large-scale fading and delayed CSI), justified by vehicle proximity. Together with the fact that the two directions’ small-scale fading components share the same distribution (unit-mean exponential), this symmetry implies that $\mathrm{Pr}\left\{\gamma_{i_{1}}^{\mathrm{FD},d}\leq \gamma_{0}^{d}\right\}=\mathrm{Pr}\left\{\gamma_{i_{2}}^{\mathrm{FD},d}\leq \gamma_{0}^{d}\right\}$. Hence, the symmetric sufficient condition for C2 is

(19) $$\mathrm{Pr}\left\{\gamma_{i}^{\mathrm{FD},d}\leq \gamma_{0}^{d}\right\}\leq p_{1},$$

where the threshold $p_{1}$ is determined by the residual correlation between the two directional outage events. Let $q=\mathrm{Pr}\left\{\gamma_{i_{1}}^{\mathrm{FD},d}\leq \gamma_{0}^{d}\right\}=\mathrm{Pr}\left\{\gamma_{i_{2}}^{\mathrm{FD},d}\leq \gamma_{0}^{d}\right\}$ denote the marginal outage probability of each direction. The joint outage probability can be expressed as $\mathrm{Pr}\left\{\gamma_{i_{1}}^{\mathrm{FD},d}\leq \gamma_{0}^{d}\cap \gamma_{i_{2}}^{\mathrm{FD},d}\leq \gamma_{0}^{d}\right\}=q^{2}+\rho \;q\left(1-q\right)$, where ρ∈[0,1] is the correlation coefficient reflecting the residual correlation of bidirectional fast fading; ρ=0 corresponds to full independence and ρ=1 to full correlation. Solving the joint outage constraint for *q* yields

(20) $$p_{1}\left(\rho \right)=\frac{-\rho +\sqrt{\rho^{2}+4\left(1-\rho \right)p_{0}}}{2(1-\rho )},\;\rho \in \left[0,1\right).$$

The threshold $p_{1}$ satisfies $p_{0}\leq p_{1}\leq \sqrt{p_{0}}$. Specifically, ρ=0 (fully independent) gives $p_{1}=\sqrt{p_{0}}$, the most relaxed threshold and the most favorable condition for FD; ρ=1 (fully correlated) gives $p_{1}=p_{0}$, the most stringent threshold; and 0<ρ<1 yields $p_{0}<p_{1}<\sqrt{p_{0}}$. Assuming full independence (ρ=0) is a standard practice in FD bidirectional communication systems: for example, Lu et al. [27] assumed independent and identically distributed forward and backward channels in IRS-assisted FD two-way communications, and a similar assumption is adopted in [28]. In practice, ρ can be estimated from pilot measurements of the bidirectional links, e.g., as the sample correlation coefficient of the measured channel-gain sequences of the two directions, and configured at the BS as a semi-static parameter; when such measurements are unavailable, the conservative choice ρ=1 (i.e., $p_{1}=p_{0}$) guarantees reliability. The SINRs in Equations (4) and (5) can be written as

(21) $$\gamma_{i}^{\mathrm{FD},d}=\frac{E+FX}{G+HY},$$

where $X=|e_{i}{|}^{2}/\left(1-\epsilon_{i}^{2}\right)$ and $Y=|e_{j,i}{|}^{2}/\left(1-\epsilon_{j,i}^{2}\right)$ are independent unit-mean exponential random variables. The outage event from Equation (21) can be rewritten as

(22) $$FX-H\gamma_{0}^{d}Y\leq G\gamma_{0}^{d}-E.$$

Therefore, based on Equation (22), the integration domain depends on the sign of $G\gamma_{0}^{d}-E$, which divides the feasible region into two cases. If $G\gamma_{0}^{d}\geq E$, then the upper limit of *X* is non-negative for all Y≥0. As shown in Equation (23),

(23) $$\begin{array}{c} \mathrm{Pr}\left\{\gamma_{i}^{\mathrm{FD},d}\leq \gamma_{0}^{d}\right\}=\int_{0}^{\infty }e^{-y}dy\int_{0}^{\frac{\left(G+Hy\right)\gamma_{0}^{d}-E}{F}}e^{-x}dx=1-\frac{\exp\left(\frac{E-G\gamma_{0}^{d}}{F}\right)}{1+\frac{H}{F}\gamma_{0}^{d}}\leq p_{1}, \end{array}$$

which is equivalent to Equation (17). If $G\gamma_{0}^{d}<E$, the outage event occurs only when *Y* is sufficiently large. In this case, integrating first over *Y* gives

(24) $$\begin{array}{c} \mathrm{Pr}\left\{\gamma_{i}^{\mathrm{FD},d}\leq \gamma_{0}^{d}\right\}=\int_{0}^{\infty }e^{-x}dx\int_{\frac{E+Fx-G\gamma_{0}^{d}}{H\gamma_{0}^{d}}}^{\infty }e^{-y}dy=\frac{\exp\left(\frac{G\gamma_{0}^{d}-E}{H\gamma_{0}^{d}}\right)}{1+\frac{F}{H\gamma_{0}^{d}}}\leq p_{1}. \end{array}$$

Rearranging Equation (24) yields Equation (18). Combining the two cases with the peak power constraints C3 and C4 completes the proof. □

It is noted that the symmetric sufficient condition in Equation (19) is a conservative relaxation of the original constraint C2 rather than an exact reformulation. When the two marginal outage probabilities are equal, the condition is tight; otherwise, it may exclude feasible pairs where one direction’s outage probability exceeds $p_{1}$ while their product still satisfies $p_{0}$. This conservative design is justified by the statistical symmetry of the bidirectional links and the QoS fairness requirement for V2V communication.

Based on the above lemmas, the optimal power solution for P3 is provided in Theorem 1.

**Theorem 1.**  The optimal power solution $\left(P_{i}^{\mathrm{FD},d^{*}},P_{j}^{\mathrm{FD},c^{*}}\right)$ for the CUV_*j*_–DUV_*i*_ pair under FD-V2V sharing mode is given by:

(25) $$P_{i}^{\mathrm{FD},d^{*}}=\left\{\begin{array}{cc} \min\left\{P_{\max}^{d},\;f_{1}^{-1}\left(P_{\max}^{c}\right)\right\}, & P_{\max}^{d}\leq P_{0}^{d},\\ \min\left\{P_{\max}^{d},\;f_{2}^{-1}\left(P_{\max}^{c}\right)\right\}, & P_{\max}^{d}>P_{0}^{d},\;P_{\max}^{c}>P_{0}^{c},\\ f_{1}^{-1}\left(P_{\max}^{c}\right), & P_{\max}^{d}>P_{0}^{d},\;P_{\max}^{c}\leq P_{0}^{c}. \end{array}\right.$$

(26) $$P_{j}^{\mathrm{FD},c*}=\left\{\begin{array}{cc} \min\left\{P_{\max}^{c},\;f_{1}\left(P_{\max}^{d}\right)\right\}, & P_{\max}^{d}\leq P_{0}^{d},\\ \min\left\{P_{\max}^{c},\;f_{2}\left(P_{\max}^{d}\right)\right\}, & P_{\max}^{d}>P_{0}^{d},\;P_{\max}^{c}>P_{0}^{c},\\ P_{\max}^{c}, & P_{\max}^{d}>P_{0}^{d},\;P_{\max}^{c}\leq P_{0}^{c}. \end{array}\right.$$

*$P_{0}^{c}$ and $P_{0}^{d}$ are calculated by Equation (*27*),*

(27) $$\begin{array}{c} P_{0}^{c}=\frac{\sigma^{2}}{\begin{array}{cc} \; & \frac{1-\epsilon_{j,i}^{2}}{1-\epsilon_{i}^{2}}\cdot \frac{1-p_{1}}{p_{1}}\cdot \alpha_{j,i}\left(\epsilon_{i}^{2}{\left|{\hat{h}}_{i}^{\prime }\right|}^{2}-\frac{\eta \gamma_{0}^{d}}{\alpha_{i}}\right)-\alpha_{j,i}\epsilon_{j,i}^{2}{\left|{\hat{h}}_{j,i}^{\prime }\right|}^{2} \end{array}},\\ P_{0}^{d}=\frac{P_{0}^{c}\left(1-p_{1}\right)\gamma_{0}^{d}\alpha_{j,i}\left(1-\epsilon_{j,i}^{2}\right)}{p_{1}\alpha_{i}\left(1-\epsilon_{i}^{2}\right)}, \end{array}$$

*where $f_{1}\left(\cdot \right)$ and $f_{2}\left(\cdot \right)$ are the explicit expressions of the implicit functions $F_{1}\left(P_{i}^{d},P_{j}^{c}\right)=0$ and $F_{2}\left(P_{i}^{d},P_{j}^{c}\right)=0$, respectively. When $P_{i}^{d}\in \left(0,P_{0}^{d}\right)$, the upper boundary of the feasible region is $F_{1}=0$; when $P_{i}^{d}\in \left(P_{0}^{d},+\infty \right)$, it becomes $F_{2}=0$. For the optimal power solutions in Equations (*25*) and (*26*) to be valid, the channel parameters must satisfy the following constraints:*

(28) $$\left\{\begin{array}{c} P_{\max}^{d}\alpha_{i}\epsilon_{i}^{2}{\left|{\hat{h}}_{i}^{\prime }\right|}^{2}-\left(P_{\max}^{d}\eta +\sigma^{2}\right)\gamma_{0}^{d}\geq 0,\\ \alpha_{i}\epsilon_{i}^{2}{\left|{\hat{h}}_{i}^{\prime }\right|}^{2}-\eta \gamma_{0}^{d}\geq 0. \end{array}\right.$$

**Remark on the constraint in Equation **(28)**:** The constraint in Equation (28) is a sufficient channel condition for the closed-form solution in Theorem 1. It guarantees that the feasible region induced by the V2V outage constraint is nonempty and that the active upper boundary has the monotonicity required by the proof. Physically, it requires the useful V2V signal, under delayed CSI uncertainty, to dominate residual self-interference and noise, thereby imposing a requirement on the SIC capability η. Although this condition can be relaxed, the compact closed-form rule in Theorem 1 would no longer be guaranteed; boundary points or stationary points must then be searched numerically for each CUV–DUV pair, which is less convenient for engineering implementation.

**Proof.**  Let $R(P_{i}^{d},P_{j}^{c})$ denote $R_{i,j}^{\mathrm{FD},c}$, $S_{j}=\alpha_{j,B}h_{j,B}$, $I_{i}=\alpha_{i_{1},B}h_{i_{1},B}+\alpha_{i_{2},B}h_{i_{2},B}$, $B_{i}=\sigma^{2}+P_{i}^{d}I_{i}$, and $D_{i}=B_{i}+P_{j}^{c}S_{j}$. From the original SINR expression, for any feasible point with positive transmit powers,

(29) $$\begin{array}{cc} \frac{\partial R}{\partial P_{j}^{c}} & =E\;\left[\frac{S_{j}}{\ln2\;D_{i}}\right]>0,\\ \frac{\partial R}{\partial P_{i}^{d}} & =-E\;\left[\frac{P_{j}^{c}S_{j}I_{i}}{\ln2\;B_{i}D_{i}}\right]<0. \end{array}$$

Hence, no interior point with a feasible increase in $P_{j}^{c}$ can be optimal. The optimum must be located on the upper boundary imposed by the V2V outage constraint or the peak-power boundary.

According to Lemma 2, the upper boundary is divided by $G\gamma_{0}^{d}=E$ into two segments. After taking logarithms in Equations (17) and (18), these two active boundaries can be written as

(30) $$\begin{array}{cc} F_{1}\left(P_{i}^{d},P_{j}^{c}\right) & =\frac{G\gamma_{0}^{d}-E}{F}+\ln\;\left(1+\frac{H\gamma_{0}^{d}}{F}\right)+\ln\left(1-p_{1}\right)=0,\\ F_{2}\left(P_{i}^{d},P_{j}^{c}\right) & =\ln\;\left(1+\frac{F}{H\gamma_{0}^{d}}\right)+\frac{E-G\gamma_{0}^{d}}{H\gamma_{0}^{d}}+\ln p_{1}=0. \end{array}$$

The intersection of the two boundary segments and the separating line $G\gamma_{0}^{d}=E$ gives $\left(P_{0}^{d},P_{0}^{c}\right)$ in Equation (27). By the implicit function theorem, the slopes of the two boundary segments are

(31) $$\frac{dP_{j}^{c}}{dP_{i}^{d}}=-\frac{\partial F_{\ell }/\partial P_{i}^{d}}{\partial F_{\ell }/\partial P_{j}^{c}},\;\ell \in \left\{1,2\right\}.$$

For $F_{1}=0$, direct differentiation of Equation (30) yields $\partial F_{1}/\partial P_{j}^{c}>0$ and $\partial F_{1}/\partial P_{i}^{d}<0$ on $0<P_{i}^{d}<P_{0}^{d}$, and therefore $f_{1}\left(P_{i}^{d}\right)$ is strictly increasing. For $F_{2}=0$, this monotonicity is not unconditional; the constraint in Equation (28) is imposed as a sufficient channel condition under which $\partial F_{2}/\partial P_{j}^{c}>0$ and $\partial F_{2}/\partial P_{i}^{d}<0$, so $f_{2}\left(P_{i}^{d}\right)$ is also strictly increasing on $P_{i}^{d}>P_{0}^{d}$.

It remains to show that increasing along this upper boundary improves the objective. Parameterizing either boundary segment by $P_{i}^{d}$ gives, as shown in Equation (32),

(32) $$\frac{dR}{dP_{i}^{d}}=\frac{\partial R}{\partial P_{i}^{d}}+\frac{\partial R}{\partial P_{j}^{c}}\frac{dP_{j}^{c}}{dP_{i}^{d}}.$$

Substituting Equations (29) and (31) shows that $\frac{dR}{dP_{i}^{d}}>0$ is equivalent to

(33) $$\frac{dP_{j}^{c}}{dP_{i}^{d}}>-\frac{\partial R/\partial P_{i}^{d}}{\partial R/\partial P_{j}^{c}}.$$

The right-hand side of Equation (33) is positive and finite. Under the constraint in Equation (28), differentiating the active outage constraint gives exactly this slope dominance on both boundary segments. Therefore, *R* is strictly increasing along the feasible upper boundary. The optimal point is consequently the feasible upper-boundary point with the largest $P_{j}^{c}$ after applying $P_{j}^{c}\leq P_{\max}^{c}$ and $P_{i}^{d}\leq P_{\max}^{d}$, which yields the three cases stated in Theorem 1. □

Substitute the optimal power solution into Equation (10) and verify whether the QoS requirements are satisfied, thereby obtaining the optimal ergodic rate $R_{i,j}^{\mathrm{FD},c*}$ of the CUV in the sharing pair.

### 3.2. Spectrum Allocation for Multi-User

After computing $R_{i,j}^{\mathrm{FD},c*}$ for all potential sharing pairs following the approach for solving P3, the original problem P1 of maximizing the system’s sum rate can be simplified to the optimization problem P4 in Equation (34),

(34) $$\begin{array}{cc} P4: & \max_{\rho_{i,j}}\sum_{j=1}^{J}\sum_{i=1}^{J}\rho_{i,j}R_{i,j}^{\mathrm{FD},c*}\\ s.t. & \mathrm{Equations}\;(7f)\;\mathrm{and}\;(7g), \end{array}$$

where I≤J, thus requiring the addition of J−I virtual DUVs with zero power. The values $\left\{R_{i,j}^{\mathrm{FD},c*}|j\in C,i\in \left[I+1,J\right]\right\}$ can be calculated using Equation (6). Clearly, P4 constitutes a bipartite matching problem that can be solved using the Kuhn–Munkres (KM) algorithm applied to the square matrix ${\left\{R_{i,j}^{\mathrm{FD},c*}\right\}}_{J\times J}$, thereby obtaining the maximum system sum rate. Since the MaxSum problem directly reduces to a standard maximum-weight bipartite matching problem, its procedure is straightforward and is therefore described in text rather than listed as a separate algorithm.

Similarly, the original maximin problem P2 can be simplified to the problem P5 in Equation (35):

(35) $$\begin{array}{cc} P5:\;\;\;\; & \max_{\rho_{i,j}}\min_{j\in C}\sum_{i=1}^{J}\rho_{i,j}R_{i,j}^{\mathrm{FD},c^{*}}\\ s.t.\;\;\;\; & \mathrm{Equations}\;(7f)\;\mathrm{and}\;(7g). \end{array}$$

To solve P5, the binary-search-based maximization bottleneck value algorithm is applied to the matrix ${\left\{R_{i,j}^{\mathrm{FD},c*}\right\}}_{J\times J}$. The overall idea is to perform a binary search over the sorted rate entries: for each candidate threshold, a judging matrix E is constructed whose entries indicate whether each pair’s rate meets the threshold, and the Hungarian algorithm is invoked to check whether a perfect matching exists among the feasible entries. The procedure is summarized in Algorithm 1.

The binary search in Algorithm 1 relies on the following feasibility observation. For a candidate threshold $s_{m}$, the judging matrix E marks the entries whose rates are lower than $s_{m}$, and the minimum total weight *w* returned by the Hungarian algorithm indicates feasibility: w=0 means that an assignment exists in which no selected entry of $R^{\mathrm{FD}}$ is smaller than $s_{m}$, whereas w>0 means that every assignment must select at least one entry below $s_{m}$. The binary search updates the lower or upper index accordingly and finally returns the spectrum sharing matrix ρ and the maximized minimum CUV rate $R_{\min}^{*}$. The binary search requires $O\left(\log\left(J^{2}\right)\right)=O\left(\log J\right)$ iterations, each invoking the Hungarian algorithm with $O(J^{3})$ complexity, yielding an overall complexity of $O(J^{3}\log J)$.

### 3.3. Mode Selection for AFD

Following the method for computing the matrix ${\left\{R_{i,j}^{\mathrm{FD},c*}\right\}}_{J\times J}$ in FD-V2V mode, the ergodic rate matrix ${\left\{R_{i,j}^{\mathrm{HD},c*}\right\}}_{J\times J}$ for HD-V2V mode can be obtained. Comparison reveals that elements in the FD matrix are not always superior to those in the HD matrix; under certain channel conditions, stronger interference in FD mode leads to power constraints that degrade the CUV’s rate compared to HD mode. Thus, by incorporating optimal duplex mode selection into the spectrum allocation, we propose the AFD scheme to further improve system performance in both sum rate and minimum rate. The AFD ergodic rate matrix ${\left\{R_{i,j}^{\mathrm{AFD},c^{*}}\right\}}_{J\times J}={\left\{\max\left(R_{i,j}^{\mathrm{FD},c^{*}},R_{i,j}^{\mathrm{HD},c^{*}}\right)\right\}}_{J\times J}$ is then employed in the spectrum allocation procedure. For simplicity, the sum rate maximization algorithm is denoted as MaxSum and the minimum rate maximization algorithm as MaxMin.

**Remark on opportunistic FD activation:** In practical deployment, the initial working mode of each terminal vehicle is set to HD. In each resource reallocation period, the BS checks whether the current channel conditions satisfy the constraint in Equation (28). If satisfied, the BS triggers the resource allocation algorithm and signals the terminal to activate FD mode; otherwise, the vehicle remains in HD mode. This opportunistic activation is consistent with the adaptive FD (AFD) philosophy: FD is not enforced but selectively enabled when channel conditions permit.

## 4. Numerical Results

This section presents numerical simulation results to validate the superiority of the proposed AFD interference management algorithm. The default values of the main parameters are listed in Table 3, while the settings in the figures take precedence. Other parameters follow the V2I and V2V path loss models referenced in [2].

The parameter settings in Table 3 are justified as follows. The deployment and RF parameters (carrier frequency, bandwidth, transmit powers, lane width, and BS-to-road distance) follow the highway evaluation scenario in 3GPP TR 36.885 [29], and the traffic model with the velocity-dependent average inter-vehicle distance follows [2]. The delayed-CSI parameters, i.e., the FD delay coefficient κ and the default feedback delay T=1 ms on the order of the 5G subframe duration, follow [5]. The SIC capability |η|=110 dB is adopted because such a cancellation level is commonly achieved in practical FD implementations [6] and thus represents a realistic operating point. Finally, $p_{0}=0.1\%$ (99.9% reliability), $\gamma_{0}^{d}=5$ dB, and $R_{0}^{c}=0.5$ bps/Hz are typical requirements of V2V safety and cellular services.

The outage threshold is set to $p_{1}=\sqrt{p_{0}}$ (i.e., ρ=0), corresponding to the ideal case of independent bidirectional fading. As analyzed in Section 2, the actual threshold lies in $\left[p_{0},\sqrt{p_{0}}\right]$ depending on the channel correlation; the independent case yields the most relaxed feasible region and thus represents an upper bound on system performance.

Three schemes are compared under identical conditions: all use the same delayed CSI, the same power-control and pairing procedures, and satisfy the same constraints C1–C6, differing only in duplex mode. FD-V2V (resp. HD-V2V) fixes all pairs in FD (resp. HD) mode, while AFD-V2V selects, for each pair, the mode with the higher rate. Unless otherwise specified, all the performance figures use 1000 independent Monte Carlo channel realizations.

This paper evaluates the duplex-mode-based MaxSum algorithm using the ergodic sum rate $\sum R_{j}$ and assesses the MaxMin algorithm using the minimum ergodic rate $\min R_{j}$. Figure 2 shows the system performance variation curves with respect to CSI feedback delay. It can be observed that all the duplex modes exhibit similar trends under both metrics, with performance degrading as CSI delay increases. Furthermore, higher velocity leads to more severe performance degradation with increasing delay. In comparison, FD and AFD schemes are less affected by delay variations, benefiting from FD’s bidirectional transmission property that reduces latency. Additionally, the AFD scheme demonstrates significant performance improvement over the HD scheme, indicating the complementary nature of FD and HD: FD outperforms HD under certain channel conditions, while the opposite holds under others. At T=1 ms and v=60 km/h, AFD-V2V achieves an approximately 13% higher ergodic sum rate and 36% higher minimum ergodic rate than HD-V2V.

Figure 3 further illustrates the impact of vehicle velocity under two CSI feedback delays. For both the ergodic sum rate and the minimum ergodic rate, all the schemes degrade as velocity increases because faster channel variation reduces the correlation between the delayed CSI and the current channel state. The performance loss is more evident when the feedback delay increases from T=0.8 ms to T=1.6 ms, especially for HD-V2V. In contrast, FD-V2V and AFD-V2V are more robust to high mobility due to the shorter effective CSI reporting delay in FD mode. Moreover, AFD-V2V consistently achieves the best performance by adaptively selecting the better duplex mode, and its advantage becomes more pronounced at high velocity and large feedback delay. At T=0.8 ms and v=120 km/h, AFD-V2V achieves approximately 14% higher ergodic sum rate and 42% higher minimum ergodic rate than HD-V2V.

Figure 4 shows the performance of different duplex modes versus SIC capability with and without the constraint in Equation (28). For Figure 2 and Figure 3, all the channel realizations are filtered to satisfy the constraint in Equation (28), so Theorem 1 provides theoretically optimal power allocation. In Figure 4, an additional set of curves without the constraint in Equation (28) is included to illustrate its impact. Under the constraint in Equation (28), the FD scheme outperforms HD over the entire |η| range (|η|≥65dB) but with limited gains; the AFD scheme achieves more significant improvements—approximately 12% higher ergodic sum rate and 36% higher minimum ergodic rate than HD-V2V at |η|=110 dB and v=75 km/h. Without Equation (28), both $\sum R_{j}$ and $\min R_{j}$ drop sharply for FD at low SIC due to the loss of monotonicity in $F_{2}\left(P_{i}^{d},P_{j}^{c}\right)=0$, leading to suboptimal power allocation. In this regime, the closed-form solution of Theorem 1 is no longer theoretically optimal and serves as a heuristic. However, the performance gap between the heuristic and the true optimum is marginal, as confirmed by the small difference between the constrained and unconstrained curves. In contrast, unconstrained AFD maintains notable gains even at low |η|, indicating that FD retains advantages under some channel conditions despite overall degradation, highlighting strong complementarity for FD and HD. This indicates that the performance advantage of AFD arises not merely from the selective exclusion of unfavorable FD channels via the constraint in Equation (28) but from the intrinsic FD/HD adaptive switching mechanism.

In Figure 4, FD mode performance appears to converge when |η|>80dB; importantly, a similar convergence is observed at low |η|. This plateau, however, should not be misinterpreted as FD being insensitive to SIC capability. The underlying reason is that, at low |η|, a large fraction of CUV–DUV pairs fail to satisfy the sufficient channel condition in Equation (28), which dramatically reduces the number of feasible pairing candidates and compresses the attainable gain. As |η| increases, more pairs become feasible and the performance recovers. Figure 5 shows the number of discarded vehicle distribution sets incurred in obtaining 100 constraint-satisfying sets. Vehicle positions are randomly generated according to a Poisson process; a set is accepted only if all J×J=400 CUV–DUV pairs satisfy Equation (28); otherwise, it is discarded and regenerated. At low |η|, the high invalid parameter count indicates difficulty in satisfying the optimal solution conditions of the proposed algorithm, especially at higher velocity. As SIC capability improves (higher |η|), constraints are more easily satisfied; when |η|>110dB, nearly all random channels meet the conditions.

## 5. Conclusions

This paper introduced the FD bidirectional communication mechanism into V2V links, effectively reducing CSI feedback delay and enhancing reliability. By enabling V2V links to adaptively select either HD or FD mode based on channel conditions, two AFD-V2V interference management algorithms were further proposed. These algorithms are optimized for the ergodic sum rate and the minimum ergodic rate of the V2I links, respectively. The simulation results demonstrated that the performance degradation of the FD mode caused by increasing CSI delay is significantly lower than that of the HD mode. This advantage is consistent under both the sum rate and minimum rate metrics. This advantage becomes more pronounced as vehicle speed increases. The AFD scheme, which adaptively selects the optimal mode, significantly outperforms schemes using solely FD or HD mode.

Several directions are identified for future work. First, extensive field measurements of point-to-point bidirectional channels are needed to statistically determine the correlation coefficient ρ, enabling a more precise selection of the outage threshold $p_{1}$ through the mapping in Equation (20). Second, further optimization of the time-splitting ratio under finer frame structures is a promising avenue building upon the established AFD–continuous-splitting equivalence. Third, validation using real-world vehicle trajectories from traffic simulators (e.g., SUMO) or GPS datasets would strengthen practical relevance. Fourth, extending the single-cell framework to multi-cell scenarios with inter-cell interference coordination is an important step toward large-scale deployment.

## Figures and Tables

**Figure 1:**
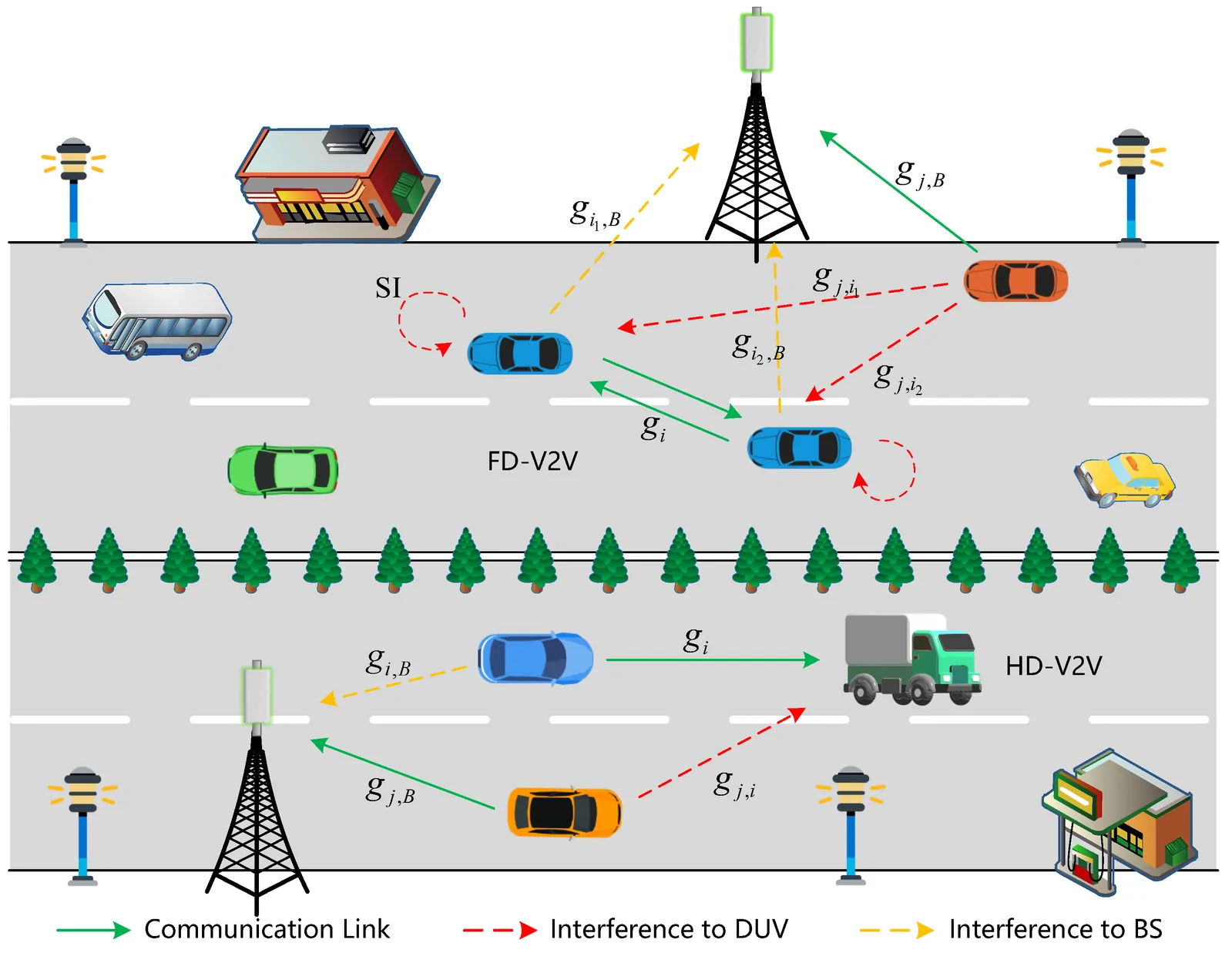
Adaptive FD-V2V and HD-V2V networking model.

**Figure 2:**
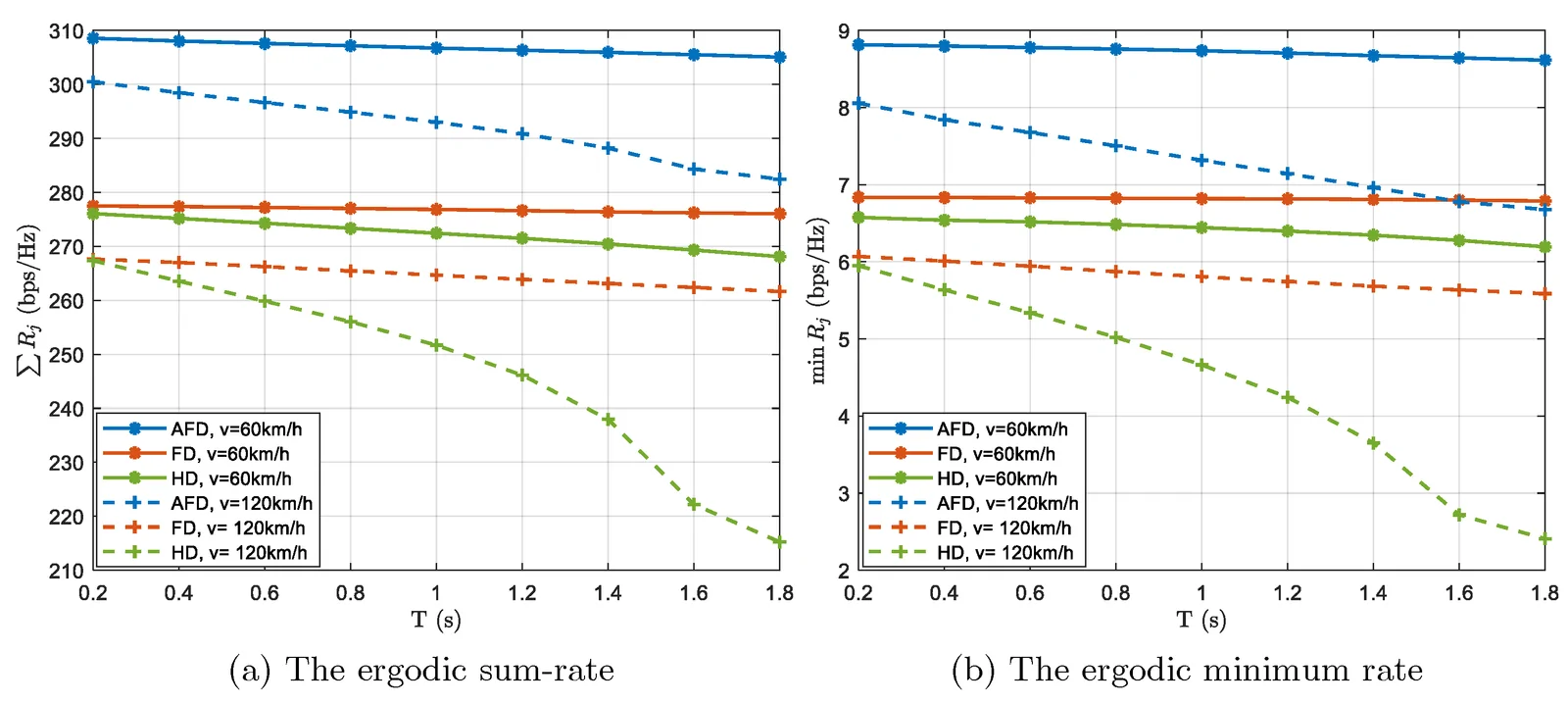
System performance comparison for various networking schemes with respect to CSI feedback delay.

**Figure 3:**
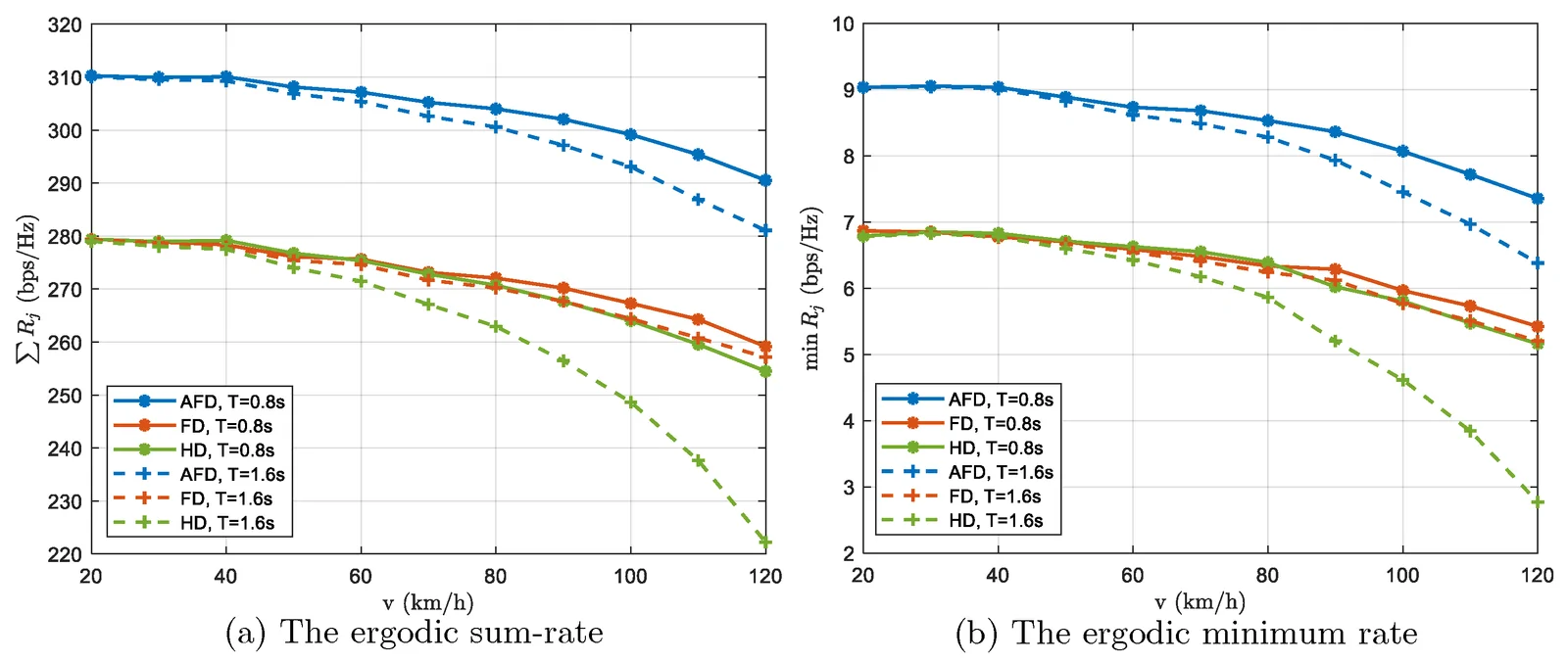
System performance comparison for various networking schemes with respect to vehicle velocity.

**Figure 4:**
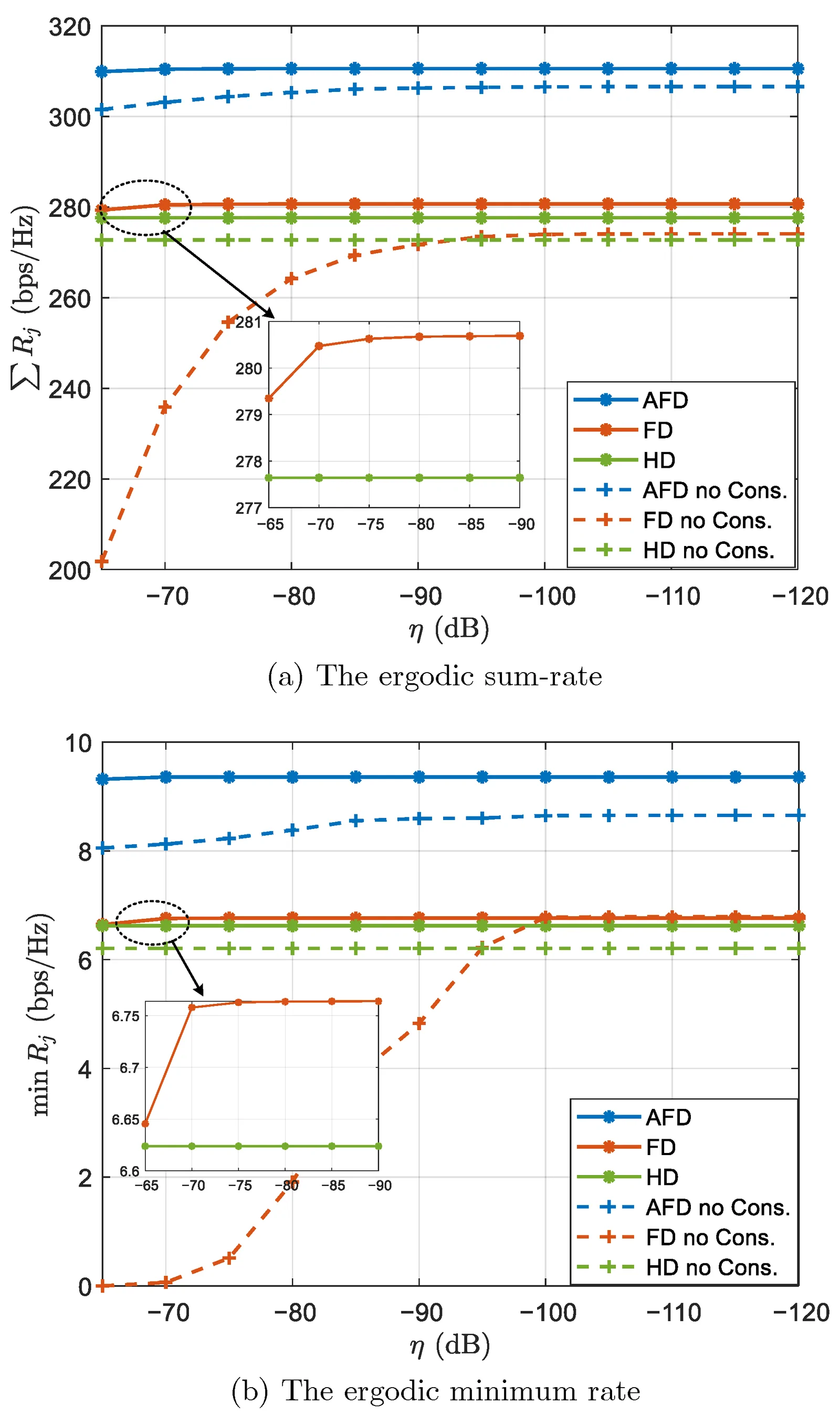
System performance comparison with respect to SIC capability.

**Figure 5:**
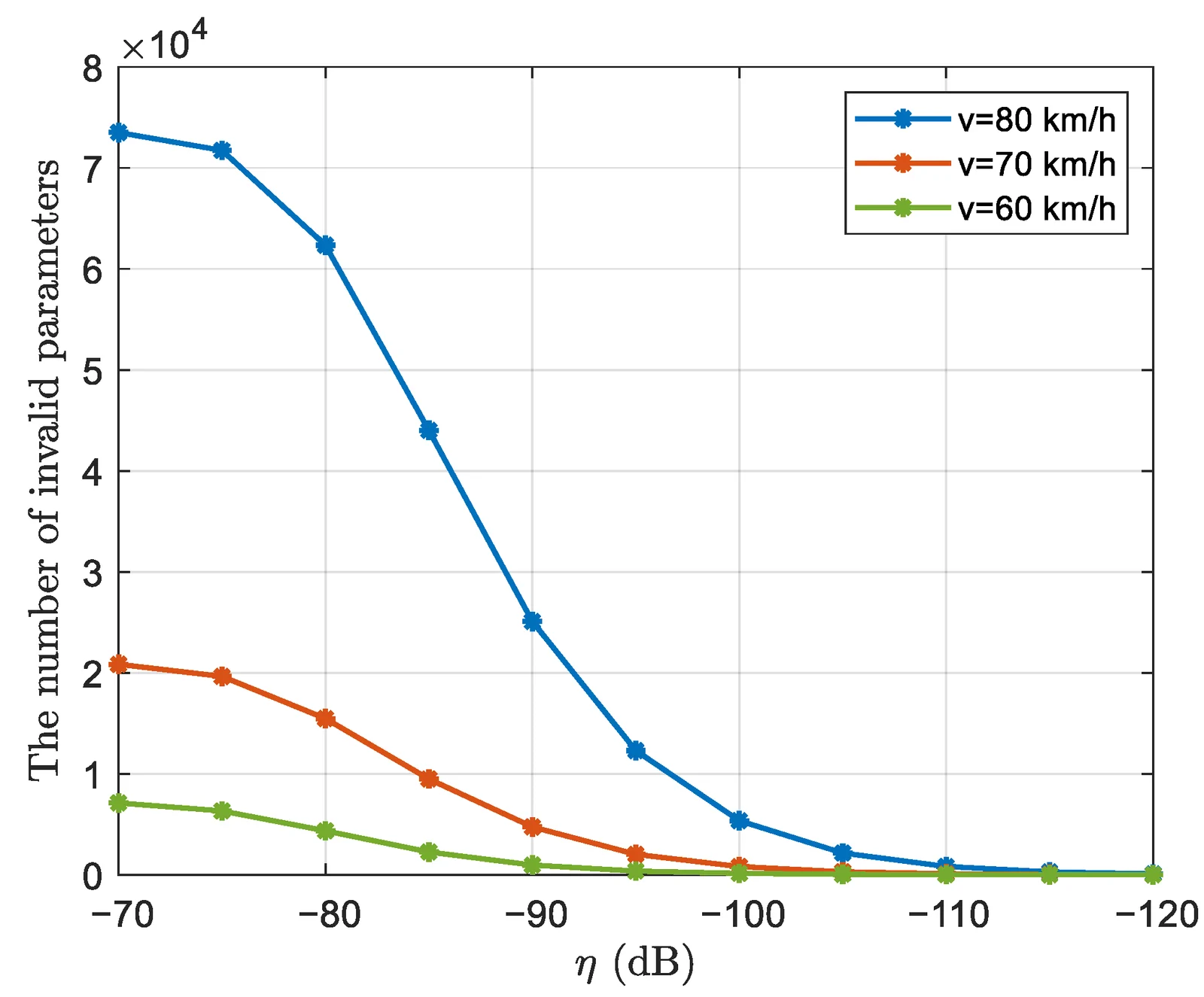
Number of discarded vehicle distribution generations incurred in obtaining 100 sets satisfying the constraint in Equation (28) with respect to SIC capability.

**Table 1:** Comparison of related works.

Ref.	Duplex	CSI Model	Objective	Solution
[2]	HD	Large-scale	Sum/min rate	Closed-form + pairing
[5]	HD	Delayed CSI	Sum rate	Closed-form + pairing
[17]	HD	Imperfect CSI	Sum rate	Joint opt.
[7]	Adaptive	Imperfect CSI	Throughput	Heuristic
[23]	Adaptive	Est. error	Spectral eff.	Heuristic
Ours	Adaptive	Delayed CSI	Sum/min rate	Closed-form + pairing

**Table 2:** Summary of main notations.

Symbol	Description
*J*, C	Number of CUVs and their index set
*I*, D	Number of DUV pairs and their index set
$i_{1}$, $i_{2}$	Transmitter and receiver of DUV pair *i*
$g_{j,B}$	Channel power gain (e.g., from CUV_*j*_ to the BS)
$h_{j,B}^{\prime }$, $h_{j,B}$	Small-scale fading amplitude and power
$\alpha_{j,B}$	Large-scale fading component
*A*, γ, $\psi_{j,B}$	Path loss constant, path loss exponent, and shadow fading variable
$d_{j,B}$	Distance between CUV_*j*_ and the BS
ϵ, *e*	Fading correlation coefficient and innovation term
*T*, $T_{\mathrm{FD}}=\kappa T$	CSI reporting periods in HD and FD modes (κ: FD delay coefficient)
$P_{j}^{c}$, $P_{i}^{d}$	Transmit powers of CUV_*j*_ and DUV_*i*_
$P_{\max}^{c}$, $P_{\max}^{d}$	Maximum transmit powers of CUVs and DUVs
η	Residual self-interference level (SIC capability)
$\sigma^{2}$	Noise power
$\rho_{i,j}$	Spectrum sharing indicator between CUV_*j*_ and DUV_*i*_
$\gamma_{j}^{\mathrm{FD},c}$, $\gamma_{i}^{\mathrm{FD},d}$	Received signal-to-interference-plus-noise ratios of the CUV and DUV links
$R_{j}^{\mathrm{FD},c}$	Ergodic rate of CUV_*j*_ in FD mode
$R_{0}^{c}$	Access rate requirement of each CUV
$\gamma_{0}^{d}$, $p_{0}$	Outage threshold and tolerable outage probability of the DUV link

**Table 3:** Major simulation parameters.

Parameter	Value
The number of CUVs (*J*)	20
The number of DUVs (*I*)	20
Bandwidth (*B*)	10 MHz
Carrier frequency ($f_{c}$)	2 GHz
Vehicle velocity (*v*)	75 km/h
SIC capability (|η|)	110 dB
CSI feedback delay (*T*)	1 ms
FD delay coefficient (κ)	0.2
DUV SINR threshold ($\gamma_{0}^{d}$)	5 dB
V2V outage probability ($p_{0}$)	0.1%
CUV access rate ($R_{0}^{c}$)	0.5 bps/Hz
Cell radius	500 m
Distance from BS to road	35 m
Maximum CUV transmit power	23 dBm
Maximum DUV transmit power	23 dBm
Noise spectral density	−174 dBm/Hz
Lane width × number	4 m × 6
Average inter-vehicle distance	2.5v/3.6 m
Monte Carlo iterations	1000

## Data Availability

No new data were created or analyzed in this study. Data sharing is not applicable to this article.

## Abbreviations

The following abbreviations are used in this manuscript:

AFD	Adaptive full-duplex
AGV	Automated guided vehicle
BS	Base station
CDF	Cumulative distribution function
CSI	Channel state information
CUV	Cellular user vehicle
D2D	Device-to-device
DUV	D2D user vehicle
FD	Full-duplex
HD	Half-duplex
IoV	Internet of vehicles
KM	Kuhn–Munkres
NOMA	Non-orthogonal multiple access
OMA	Orthogonal multiple access
QoS	Quality of service
SIC	Self-interference cancellation
SINR	Signal-to-interference-plus-noise ratio
V2I	Vehicle-to-infrastructure
V2V	Vehicle-to-vehicle
V2X	Vehicle-to-everything
